# Brazilian medical students want to learn homeopathy and acupuncture in medical schools

**DOI:** 10.1590/S1516-31802006000400013

**Published:** 2006-05-04

**Authors:** Flávio Dantas, Hagen Rampes

Teixeira's^1^ findings among medical students at Faculdade de Medicina da Universidade de São Paulo confirm our results among medical students from the United Kingdom^2^ and from all Brazilian regions,^3^ namely that they have a positive attitude towards complementary healthcare and would like to learn more about it. Ernst^4^ came to the same conclusion in a systematic review that included studies involving 2,123 medical students from European countries and the United States. Our original data from Brazil are from 1997 (with the additional inclusion of phytotherapy and hypnosis) and showed that 51% of our sample had had personal experience in using homeopathy (and 84% got positive results), but only 27% had been treated by acupuncture (93% got positive results). We found that students who stated that they knew about homeopathy and acupuncture either very well or fairly well responded mostly that these practices were extremely or very effective. 82% of the students stated that homeopathy should be available in public health services and 70% in the case of acupuncture. It has also been found that doctors in São Paulo believe it is important to know complementary medicine, and that 51% already recommend it for their patients.^5^

In teaching homeopathy and acupuncture one needs to pay special attention to impartiality and showing both sides of the situation. Medical students’ assessments after five elective courses of homeopathy at Universidade Federal de São Paulo showed that 80% of them perceived its teaching to be impartial.^6^ We hope these findings will stimulate Brazilian medical educators and heads of medical schools to establish continuous teaching of these two medical specialties in Brazil, along with phytomedicine.
